# A two-stage digestion of whole murine knee joints for single-cell RNA sequencing

**DOI:** 10.1016/j.ocarto.2022.100321

**Published:** 2022-11-24

**Authors:** Dustin M. Leale, Linan Li, Matthew L. Settles, Keith Mitchell, Lutz Froenicke, Jasper H.N. Yik, Dominik R. Haudenschild

**Affiliations:** a Department of Orthopaedic Surgery, UC Davis School of Medicine, Sacramento, CA, USA; b UC Davis Bioinformatics Core Facility, Davis, CA, USA; c UC Davis DNA Technologies and Expression Analysis Cores, Davis, CA, USA

**Keywords:** Single-cell RNA sequencing, Osteoarthritis, Whole knee joint, Tissue cross-talk, Mouse model, scRNA-seq, Single-cell RNA sequencing, OA, Osteoarthritis, UMAP, Uniform Manifold Approximation and Projection, UMI, Unique Molecular Identifier

## Abstract

**Objective:**

Single-cell RNA sequencing (scRNA-seq) is a powerful technology that can be applied to the cells populating the whole knee in the study of joint pathology. The knee contains cells embedded in hard structural tissues, cells in softer tissues and membranes, and immune cells. This creates a technical challenge in preparing a viable and representative cell suspension suitable for use in scRNA-seq in minimal time, where under-digestion may exclude cells in hard tissues, over-digestion may damage soft tissue cells, and prolonged digestion may induce phenotypic drift. We developed a rapid two-stage digestion protocol to overcome these difficulties.

**Design:**

A two-stage digest consisting of first collagenase IV, an intermediate cell recovery, then collagenase II on the remaining hard tissue. Cells were sequenced on the 10x Genomics platform.

**Results:**

We observed consistent cell numbers and viable single cell suspensions suitable for scRNA-seq analysis. Comparison of contralateral knees and separate mice showed reproducible cell yields and gene expression patterns by similar cell-types. A diverse collection of structural and immune cells were captured with a majority from immune origins. Two digestions were necessary to capture all cell-types.

**Conclusions:**

The knee contains a diverse mixture of stromal and immune cells that may be crucial for the study of osteoarthritis. The two-stage digestion presented here reproducibly generated highly viable and representative single-cell suspension for sequencing from the whole knee. This protocol facilitates transcriptomic studies of the joint as a complete organ.

## 1 Introduction

Osteoarthritis (OA) is the most common degenerative joint disease affecting 30%–50% of people over 65 years old. OA is a complex disease of the whole joint, involving different joint tissues and cell types, including cells of the synovium, cartilage, subchondral bone, meniscus, tendons, fat pad, and ligaments [1,2]. Degeneration of joint tissues is driven primarily by inflammation, through activation of immune cells, through the cross-talk between tissues, and specifically, through interactions between immune cells and the different structural cells [[3], [4], [5], [6]]. It is becoming increasingly clear that immune cell functions are modulated by their interactions with a variety of structural cells that express tissue-specific immune regulators and cytokine signaling molecules [7]. Historically, gene expression studies in OA mainly focused on isolation of individual cell types and rarely examine the effects of tissue cross-talk within different joint tissues. Therefore, it is important to design new studies to elucidate the interaction and cross-talk between immune and structural cells within the joint. This will provide new insight into understanding OA pathogenesis and developing effective treatments.

Conventional microarray or bulk-RNA sequencing analyses of mixed joint tissues lack the ability to determine cell type-specific gene expression. This is largely overcome by the advent of single-cell RNA Sequencing (scRNA-seq) technology, which reveals gene expression profiles of individual cells. scRNA-seq has quickly become the gold standard for defining cell states and phenotypes in many tissues. However, the joint is a unique organ as it contains both hard (bone/cartilage/ligament) and soft (synovium/fat/tendon) tissues, which create a challenge during extraction of cells embedded in different tissue matrices. Cells residing within the synovium and other “soft” matrices are readily extracted with mild digestive enzymes, while other cells deeply embedded in cartilaginous and bony “hard” matrices require much harsher digestion with more aggressive collagenases that could damage soft tissue cells [[8], [9], [10]]. Thus, conventional single-step methods for cell extraction used in other organs/tissues, such as liver or lungs, are not suitable for joint tissues.

In this study, we developed a sequential two-step digestion protocol to isolate representative cells from all tissues in whole mouse joints. Cells embedded in soft tissues were first extracted and removed under mild enzymatic conditions, while the remaining cells embedded in hard tissues were subsequently extracted under aggressive enzymatic conditions designed for bony tissues. Digestive enzyme concentrations were optimized to minimize time, stress response, and transcriptional shift during processing. The isolated cells were then analyzed by scRNA-seq to determine individual gene expression profiles specific for known cell types found within the joint.

## 2 Materials and method

### 2.1 Mice

Adult male C57BL6 mice (The Jackson Laboratory, Bar Harbor, ME, US) aged 23–25 weeks were used in this study. All animal procedures were approved by the Institutional Animal Care and Use Committee (IACUC) at the University of California, Davis. No statistical method was used to predetermine sample size. No experiments were performed on living mice.

### 2.2 Microdissection

Immediately following euthanasia with carbon dioxide asphyxiation, mice were submerged in ice-cold 70% ethanol, and then washed in ice-cold PBS. The skin on the hind limb was removed to expose the knee joint, then the surrounding muscles were carefully dissected with scissors to expose the joint capsule. The knee joint was then excised by cutting the tibia and femur adjacent to the growth plates and transferred to a 60-mm Petri dish containing ice-cold PBS. Under a dissecting microscope, the remaining muscles were carefully trimmed until the joint capsule and growth plates were clearly visible. An incision was made along the femoral and tibial growth plates. The remaining joint tissues (typically weighting about 80 ​mg) included the femoral growth plate, ligaments, synovial lining, patella, meniscus, and the tibial growth plate, were then transferred to a dry Petri dish and minced with a scalpel. Time from animal euthanasia to minced knee tissue was approximately 10 ​minutes per knee.

### 2.3 Soft and hard tissue digestion

**Fig. 1:**
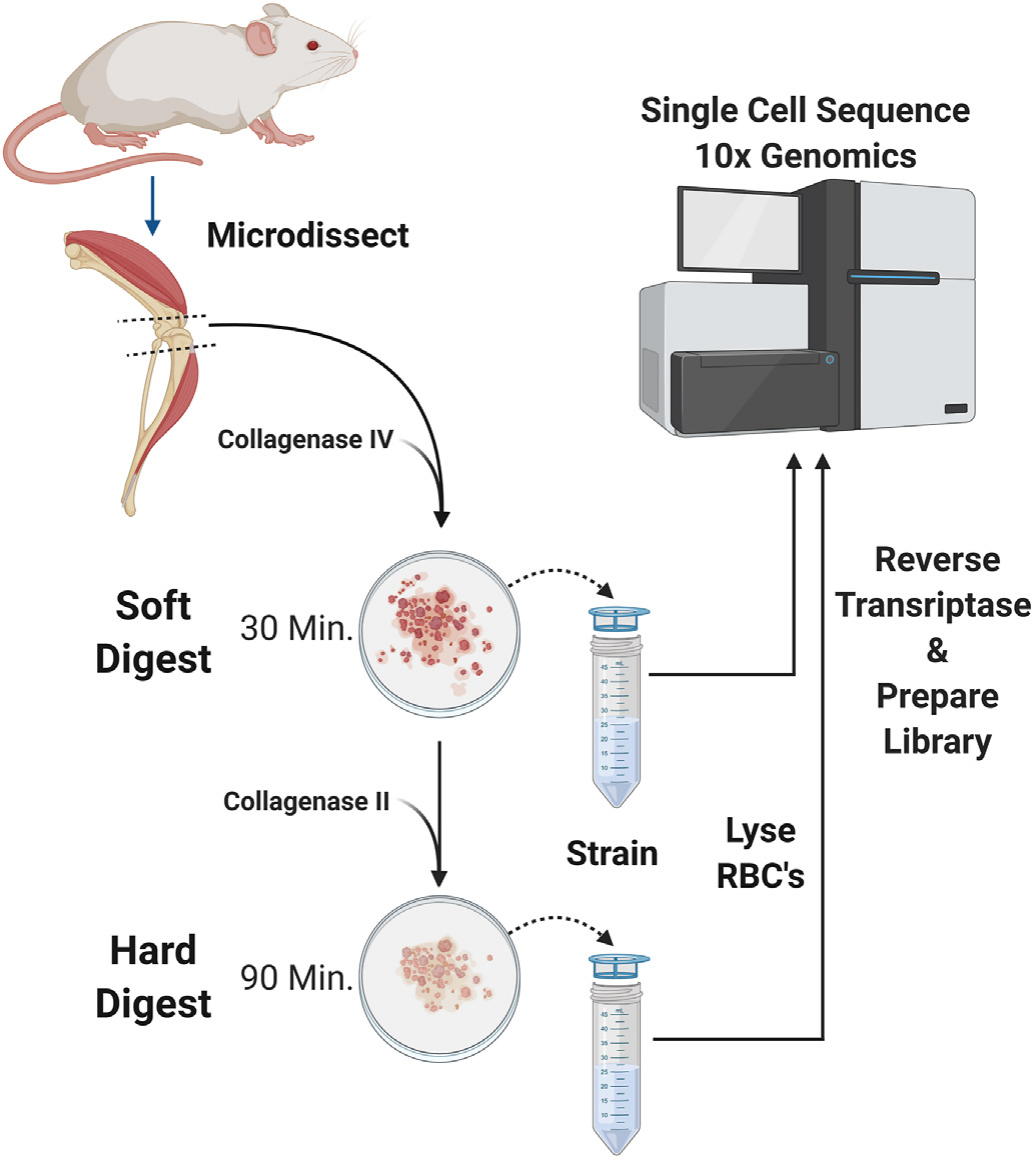
A workflow of cell isolation from soft and hard tissues for scRNA-seq. See Materials and Method for details.

The two-step digestion protocol is illustrated in Fig. 1. Briefly, the minced knee tissue was first subjected to a mild enzymatic digestion step designed to disassociate soft tissues, in 5 ​ml of 1% (w/v) collagenase IV (1600 U ​mL^−1^) (Thermo Fisher Scientific, Carlsbad, CA, US) in DMEM with 5% FBS. The soft digestion was carried out at 37 ​°C, with constant orbital rotation at 90 RPM. After 30 ​min the media containing the released cells from soft tissues was aspirated, the remaining undigested tissues were then rinsed with 10 ​ml room temperature DMEM with 5% FBS. The aspirated media and rinse media were strained twice through a 70 ​μm cell strainer (Falcon, Corning Incorporated) and further processed immediately for red blood cell lysis as described below. The remaining tissue was subjected to a second enzymatic digestion step for hard tissues, in 5 ​ml of 2% (w/v) collagenase II (2500 U ​mL^−1^) (Worthington Biochemical, Lakewood, NJ, US) in DMEM with 5% FBS. The hard tissue digestion was carried out at 37 ​°C with constant rotation for 90 ​min. At the completion of the hard digestion, cells were aspirated, rinsed, and strained as described above.

### 2.4 Red blood cell lysis

Immediately after straining, cells from soft or hard tissue digests were centrifuged at 300×*g* for 5 ​min at 4°C. To lyse the red blood cells, the cells were resuspended 10 ​ml ice-cold ACK lysis buffer (Life Technologies, Carlsbad, CA, US), followed by 10 ​minute incubation on ice following the recommendations of 10x Genomics for tissue dissociation. Cells were centrifuged and washed with 10 ​ml ice-cold DMEM/5% FBS, and resuspended in 1 ​ml ice-cold DMEM/5% FBS. The single-cell suspension was transferred on ice to the DNA Technologies and Expression Analysis Core Laboratory at UC Davis.

The entire process from both knees of one mouse was performed in less than 2.5 ​hours, resulting in two independent samples of single-cell suspension, one from soft tissue digest and one from hard tissue digest, for a total of four samples per mouse (Fig. 1).

### 2.5 Assessment of cell viability, quantity, and quality

The single-cell suspension (100 ​μl) was passed through a 40 ​μm cell strainer (Flowmi, SP Scienceware, Wayne, NJ, US) to remove debris and cellular aggregates. The samples were then visualized, and the number of live cells counted with a Luna-FL cell counter/imager (Logos Biosystems, Anyang, South Korea) under brightfield and fluorescence (Acridine Orange/Propidium Iodide) settings.

### 2.6 Library preparation and sc-RNA sequencing

Barcoded transcriptome libraries were prepared from the single-cell suspensions using the Chromium Next GEM Single Cell 3′ Kit v3.1 (10X Genomics, Pleasanton, CA, US) according to the manufacturer's instructions. Briefly, approximately 17,000 ​cells per sample were loaded onto a Chromium Next GEM Chip G and encapsulated into nanodroplets on a Chromium Controller microfluidic system, resulting in the mRNA barcoding of ∼10,000 single cells per sample. After reverse transcription, the barcoded cDNA was purified with DynaBeads (Invitrogen, Carlsbad, CA, US). After PCR amplification, the quality of the cDNA was verified by micro-capillary gel electrophoresis using a Bioanalyzer 2100 (Agilent, Santa Clara, CA, US). The amplified cDNA was fragmented and converted to 3′ gene expression profiling sequencing libraries. The fragment lengths of the sequencing libraries were assessed on a Bioanalyzer. The libraries were quantified by fluorometry on a Qubit instrument (Life Technologies, Carlsbad, CA, US) and qPCR with a Kapa Library Quant kit (Kapa Biosystems-Roche, Basel, Switzerland). Pooled libraries were sequenced on a NovaSeq 6000 sequencer (Illumina, San Diego, CA, US) with paired-end reads. The sample processing targeted 10,000 ​cells per sample and a depth of 40,000 reads per cell.

### 2.7 Data filtering

The sequencing data were processed using Cell Ranger version 5.0.1 (10X Genomics, Pleasanton, CA, US) with the mouse reference package mm10 (GENCODE vM23/Ensembl 98). To maintain the most important features of the dataset and high-quality cells, further analysis was performed only on cells expressing at least 300 genes, and only on genes that were expressed in at least 10 ​cells. Cell cycle was determined using the “scran” R-Package [11]. Mitochondrial gene expression was calculated for the data as a proportion of total gene expression per cell. Cells with greater than 90% mitochondrial gene expression (indicative of damaged cells) were excluded. A highly inclusive mitochondrial DNA threshold was chosen intentionally to visualize the distribution of mitochondrial DNA content that was observed within the joint samples. To identify possible cell-doublets in the data, we excluded cells with total genes (nFeature_RNA) that exceeded a threshold of 7800 using Seurat [12].

### 2.8 Dataset integration and cell clustering

Following the single-cell expression assignment, Seurat (Satija Lab, New York, US Version 4.0.1) was used to further analyze the data [13]. The four files output from Cell Ranger (Left knee-soft digest, left knee-hard digest, right knee-soft digest, right knee-hard digest)from each mouse were combined into one object for each mouse in Seurat for analysis going forward. Gene expression measurements were normalized with LogNormalize for each cell, multiplied by a scale factor (10,000 by default), and log-transformed. To better standardize the dataset, regression analysis was performed using the number of Unique Molecular Identifiers (UMIs), the number of genes, and the cell cycle stage. Mitochondrial gene expression was not included in the regression analysis as it may be a feature of some cell types. To combine datasets from multiple mice a set of mutually variable anchors were found between the individually processed mice datasets. These anchors were then used to integrate the two datasets using the IntegrateData() command of Seurat, and the combined dataset was then scaled. A Principal Component Analysis (PCA)-based clustering technique was performed in Seurat, at first using 50 ​PCs to be more inclusive of rare cell types [14,15]. In the final analysis presented here, 10 ​PCs were chosen to simplify presentation of the data. The final number of PCs used was determined by using an elbow, or scree plot generated by the ElbowPlot() command, and observing the point at which the y-values between points stops changing significantly. Uniform Manifold Approximation and Projection (UMAP) was chosen to display the combined datasets [16].

### 2.9 Statistical analysis

For tables one and two, means and standard deviations were calculated for multiple samples. Bioinformatics of single cell data were handled in Seurat version 4.0.1 with default values.

## 3 Results

### 3.1 Cell yield and viability

**Fig. 2:**
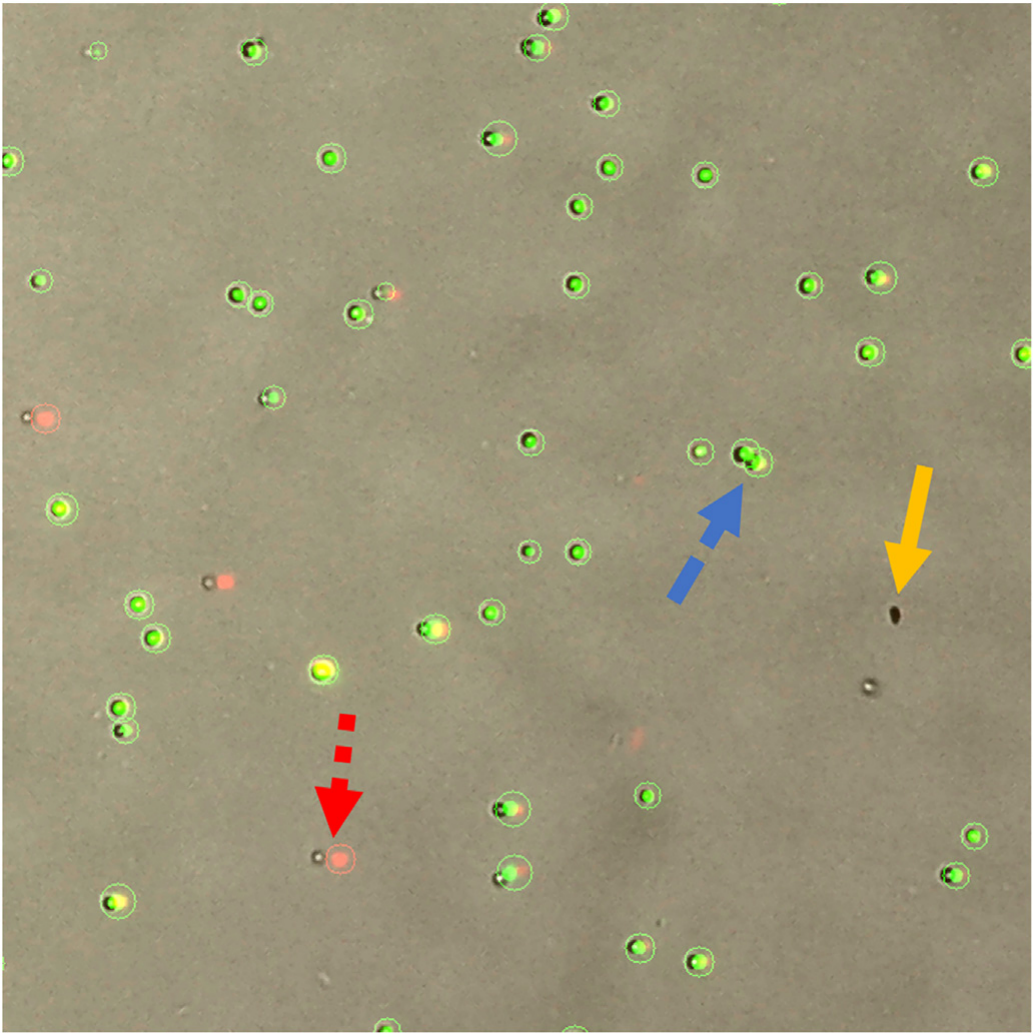
Viability and Quality of single-cell suspension. Fluorescence staining of live (Acridine Orange, green) and dead (Propidium Iodide, red) cells, overlaid on brightfield microscopy. The single-cell suspension consisted of mostly live cells (single green dots), and was mostly free of cell clusters (blue dashed arrow), dead cells (red smaller dashed arrow), and debris (orange arrow). The image from LUNA-FL imager shows an area of about 5 ​mm^2^.

The quantity and quality of cells isolated from the soft and hard tissue digestions were assessed for their suitability for scRNA-seq analysis. On average, about 6.5 million and 5 million cells were isolated in each knee from the soft tissues and hard tissues, respectively (Table 1). Cell viability was at least 87%, and the samples were mostly free of debris and cell clusters (Fig. 2). These results indicate that the two-step digestion protocol yields highly viable single-cell suspension suitable for scRNA-seq.

**Table 1:** Cell yields and viability from soft and hard joint tissues.

Soft Tissue Digest		Hard Tissue Digest	
Cell Number (Millions)	Viability (%)	Cell Number (Millions)	Viability (%)
6.5 ​± ​0.5	92.4 ​± ​0.9	4.9 ​± ​0.6	87.2 ​± ​7.2

The total number and viability of cells isolated from soft or hard knee tissues. Data reported as mean ​± ​SD from four knee digests from two mice.

### 3.2 Quality control of scRNA-seq data

**Fig. 3:**
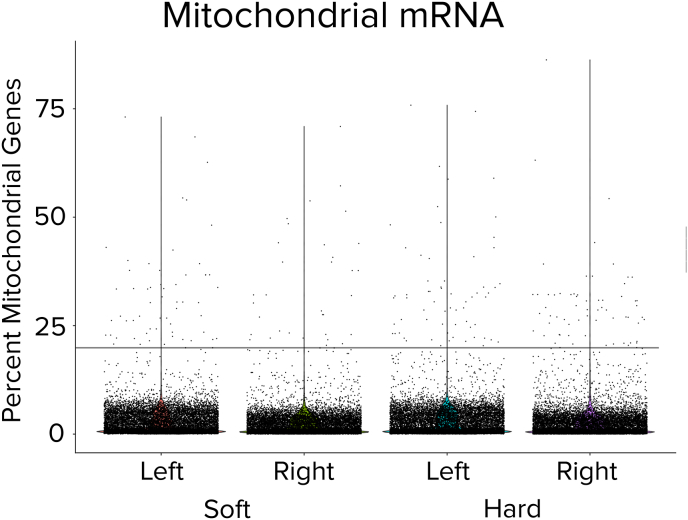
Mitochondrial DNA content of single cells. Violin plots of percent mitochondrial DNA within each digest, a line at 20% is shown as an example cut-off threshold. Each dot represented one cell. Data was obtained from sequencing four knee digests from two mice for a total of eight digests.

The quality of scRNA-seq data was assessed first by determining the mitochondrial gene content relative to total expressed genes. The mitochondrial gene profiles in all samples were comparable, regardless of the tissue origins (i.e. soft or hard tissues; left or right knees), (Fig. 3). Most of the sequenced cells within each sample were derived from cells containing less than 10% mitochondrial gene contents, an example threshold of 20% is displayed. Further, the digestion protocols yielded almost exclusively single-cells. Using a cutoff of 7800 genes/cell to indicate possible doublets, we observed only 14 ​cells out of ∼33 thousand exceeded this threshold [12]. A low mitochondrial content indicates viable cells [17], thus these results corroborated with the high viability values determined above (Fig. 2).

The quality of the scRNA-seq data was further assessed using the numeric properties of the sequencing read information with Cell Ranger. As shown in Table 2, over 8000 ​cells from each sample were sequenced to a depth of at least 40,000 reads per cell. A UMI count of at least 4000 across all samples indicated a complex library with sufficient starting materials. Thus, the depth and breadth of these sequencing data indicate that the digestion protocol yielded a high quality library with sufficient complexity for further analysis.

**Table 2:** Sequencing depth and quality control metrics.

		Number of Cells	Mean Reads per Cell	Median Genes per Cell	Median UMI Counts per Cell
Soft Digest	Left	9253 ​± ​224	49,406 ​± ​9647	1453 ​± ​28	4602 ​± ​60
	Right	8730 ​± ​224.5	57,576 ​± ​2531	1505 ​± ​32	4766 ​± ​67
Hard Digest	Left	7747 ​± ​155.5	51,084 ​± ​12,354	1306 ​± ​20	4607 ​± ​26
	Right	7984 ​± ​181	51,274 ​± ​3046	1226 ​± ​22	4023 ​± ​46

Shown were the total number of cells sequenced per sample, as well as mean reads, median genes, and unique molecular identifiers counts per cell. Data from two mice reported as averages ​± ​SD.

### 3.3 Reproducibility between contralateral knees and individual mice

**Fig. 4:**
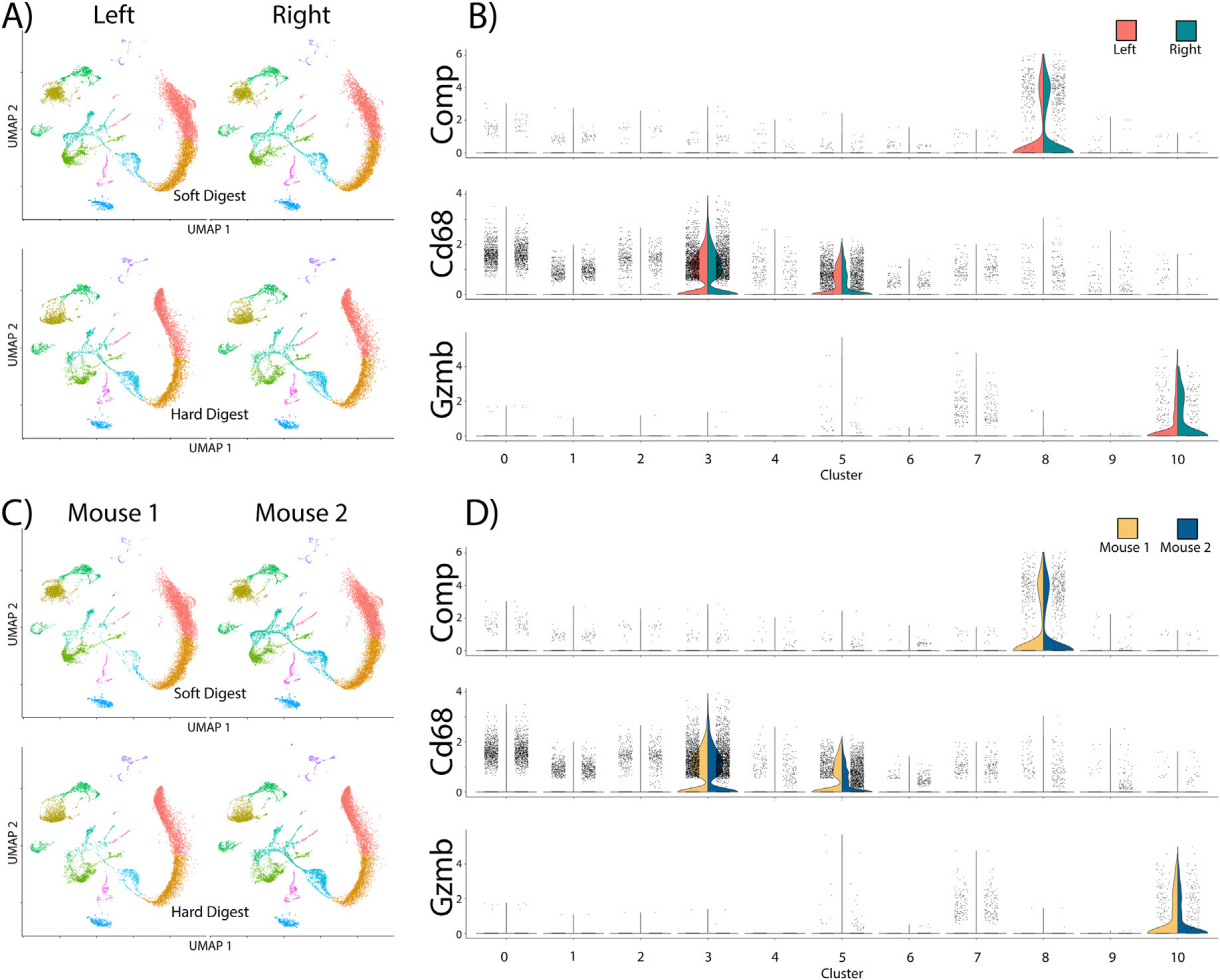
Reproducibility is demonstrated by the comparisons between contralateral knees and individual mice in both the Soft and Hard tissue digests. A) Uniform Manifold Approximation and Projection (UMAP) of single-cell RNA sequencing scRNA-seq data split by soft and hard digests from left and right knees. B) Split (Left knee/Right knee) Violin plots show expression of selected genes in different clusters. Selected genes: cartilage oligomeric matrix protein (Comp); Cd68; and Granzyme B (Gzmb). C) UMAP of single-cell RNA sequencing scRNA-seq data split by cells from soft and hard digests from Mouse 1 or Mouse 2 D) Split (Mouse 1/Mouse 2) Violin plots show expression of selected genes in different clusters. Data from two mice shown.

Reproducibility between samples is a critical aspect of protocol development. Using the combined datasets from each mouse, we compared gene expression profiles in cell samples isolated from contralateral knees and between two mice. Reproducibility is indicated by samples having similar UMAP projections and gene expression patterns (shown as 11 different clusters, each represented by a different color) (Fig. 4A). For soft tissues, data from both the left and right legs projected similar clustering and gene expression patterns (Fig. 4A). Cell populations present in the left were also present in the right, and vice versa. The same was also true for the cell populations from the hard tissues. When splitting the dataset by mouse of origin, the resulting UMAP projections are again similar (Fig. 4B). In contrast, when comparing between the soft and hard tissue digests, there were marked differences in the UMAP projections, indicating that unique cell types are isolated by the soft and hard tissue digestion protocols.

A final test of reproducibility was to determine the expression of marker genes known to be expressed in different cell-types unique or common in soft and hard tissues. The selected genes and their cellular origins are: 1) cartilage oligomeric matrix protein (Comp), chondrocytes, synovium, and other structural cells; 2) Cd68, monocytes; and 3) Granzyme B (Gzmb), Natural killer (NK) and other cytotoxic immune cells. Comp is expected to be present only in non-immune, structural tissues, whereas Cd68 and Gzmb are expected to be present in immune cells, which may include resident cells within the knee tissues. The violin plots (Fig. 4C) showed that these 3 selected marker genes were present equally in both left and right knees and equally between mice (Fig. 4D). However, each marker gene was only found in specific cell clusters. These results indicate that unique cells specific to the soft or hard tissues were reproducibly obtained from our two-step cell isolation protocol, and that both steps are required for the isolation of cells representative of all joint tissues.

### 3.4 Cell type-specific genes were present in the corresponding hard and soft tissues

**Fig. 5:**
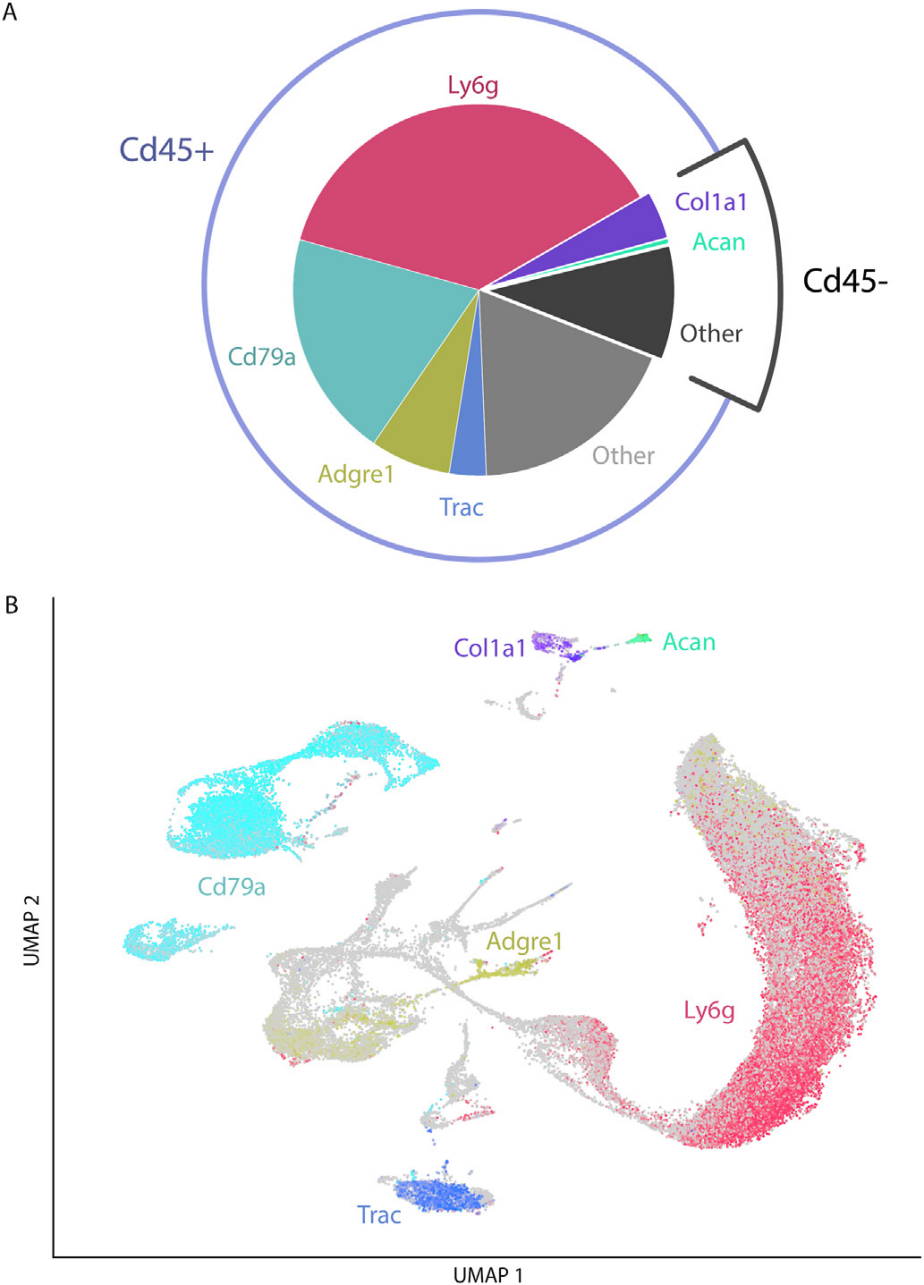
Expression of selected marker genes. A) Percentage of cells expressing selected marker genes. Cd45 expression is shown around the pie chart. Each gene was indicated by a different color: Acan (bright teal), Col1a1 (purple), Trac (blue), Adgre (gold), Ly6g (red), and Cc79a (cyan). B) Uniform Manifold Approximation and Projection of combined hard and soft tissues obtained from four knees from two mice, same color labels as above.

To determine if our digestion protocol captured representative cells from both hard and soft tissues, we used UMAP projections of the combined dataset to visualize the presence of selected marker genes for different cell types. The established marker genes and cell-types were as follows: 1) aggrecan (Acan), chondrocytes or meniscal cells; 2) Collagen type 1a (Col1a1), osteoblasts; 3) T-Cell Receptor Alpha Constant (Trac), T and NK cells; 4) Lymphocyte Antigen 6 Family Member G6D (Ly6g), monocytes and neutrophils; 5) Adhesion G Protein-Coupled Receptor E1 (Adgre1, also known as the F4/80 antigen), macrophages, and 6) Cd79a, B cells (Fig. 5a). Cd45 (Ptprc) was used to identify hematopoietic cells. To assess the cellular composition of the sample, we calculated the number of cells expressing each signature gene. When examining all cells in the combined dataset, 0.4% expressed Acan, 4.1% expressed Col1a1, 3.2% expressed Trac, 37.3% expressed Ly6g, 7% expressed Adgre1, 19.8% expressed Cd79a, and 85.1% expressed Cd45 (Fig. 5a). When these cell-type specific marker genes were visualized with the UMAP projections, each gene was present predominately within one or two clusters, indicating the presence of these different cell types (Fig. 5b).

**Fig. 6:**
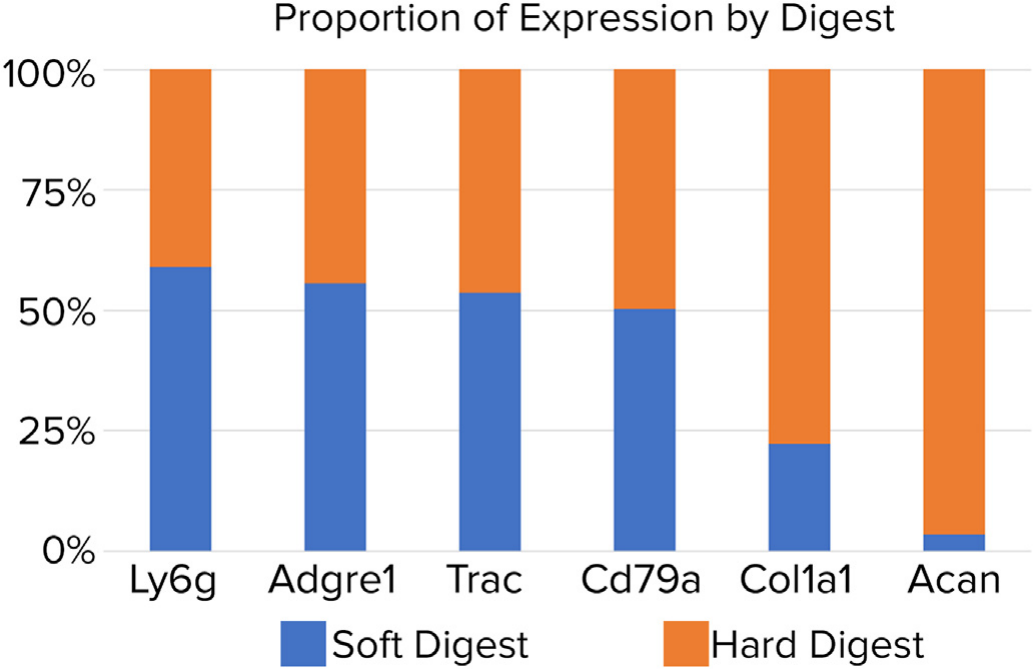
Relative expression of genes in soft and hard digests. Percentage of expression of selected genes originating from soft or hard digests. Combined data from four knees from two mice.

We next confirmed whether each marker gene originated from cell-types expected in the soft or hard tissue digests. The percentage of expression of each marker gene originating from soft or hard digest was determined (Fig. 6). Immune cell markers (Ly6g, Adgre1, Trac, and Cd79a) expected in both soft and hard tissues were distributed similarly among cells isolated from soft and hard digest. In contrast, markers of osteocytes (Col1a1) and chondrocytes (Acan) were predominant within cells isolated from the hard tissue digest (Fig. 6). Taken together, these data indicate that cell-type specific markers are found within the expected tissue types, thus validating the two-step digestion protocol for the isolation of different cell types from both hard and soft tissues.

## 4 Discussion

Recent years have seen a proliferation of scRNA-seq studies targeting specific cell populations that are isolated from different joint tissues, such as chondrocytes, synoviocytes, macrophages, and other immune cells. However, few transcriptomic studies provide a holistic approach to characterize all the different cell-types across whole joints, limiting our ability to study the physiological consequences of their tissue cross-talk. This is in part due to the presence of both soft and hard joint tissues that require different digestive enzymes for cell extraction. We developed and validated a two-step digestion protocol tailored for whole joint analysis, to isolate the tissue-specific cell types while preserving their viability (Table I, Fig. 2, Fig. 3). Analysis of scRNA-seq data with UMAP projections showed that representative cell types within their respective tissue origins were present, with high reproducibility between different cell samples isolated from different knees and different mice (Fig. 4, Fig. 5, Fig. 6).

The technology for transcriptomic analysis of mouse knee joints has evolved, from the microarray analysis in the early 2000s [18], more recently progressing to bulk RNA-seq analysis [19], and now to single-cell RNA-seq. Historically, the dissection of the mouse knee joint was performed similarly for either microarray or bulk RNA-seq analyses. The tissues analyzed generally included structures between the distal femoral growth plate and the proximal tibial growth plate. Due to the small size of each tissue in mouse joints transcriptome studies on individual tissues were generally performed in larger animal models. These whole tissue assays gave insight into the overall trends within the joint but could not identify the contributing cell-types. Several methods were developed to isolate only particular cells of interest, including the use of physical segmentation of the knee to focus on chondrocyte-rich regions [20,21], the use of cell enrichment or depletion in suspension to separate immune cells from stromal cells [19,22,23], or the culture of isolated cells [24]. Another alternative new sequencing technology is single nuclei RNA-sequencing (sNuc-Seq). This technique processes only nuclei and the RNA found within and can be performed on frozen and undigested tissues [25], however only human synovium and porcine cartilage explants have been studied this way in the joint [26,27]. Techniques used to filter or only sequence parts of the joint allow a more focused sample containing a larger proportion of “desired” cells but may miss the activities of the excluded cell populations and the crosstalk that exists within the whole joint environment. Our protocol is consistent with that used previously in microarray and bulk RNA-seq studies. Thus, the scRNA-seq data obtained from our protocol should be directly comparable to existing microarray or bulk RNA-seq data.

To our surprise, immune cells constituted the majority of cells within the whole joint digest - with 85% of cells expressing the universal hematopoietic marker Cd45. The percentage of immune cells in the knee is higher than those observed in other tissues studied by scRNA-seq [[28], [29], [30]]. In cells isolated from human lungs, 6–20% were immune cells [31]. In human skin biopsies immune cells contributed 6–40% [32]. A broad survey of human tissues using scRNA-seq found that the liver contained 45% immune cells; in the eye 31% of cells were fibroblasts and 25% were immune cells; bone marrow contained 44% NK & T cells and a large portion of B and hematopoietic stem-cells, as well as neutrophils and monocytes [33]. Among these studies in different tissues, immune cells were between 5 and 45% of all cells. A recent study using digested mouse knees found that 78.4%–70.4% of cells were Cd-45 positive using flow cytometry, with neutrophils composing the majority of those cells [34]. Cd-45 positive high immune cell component of our whole joint samples may be explained by the presence of bone marrow, which is a primary hematopoietic organ. In support of this, we found high percentages of cells expressing the universal B-cell marker Cd79a (19.4%), and Rag1 and Rag2 (4.7%) (data not shown), Rag expression takes place within the bone marrow [35]. Previous studies in the murine knee have shown Cd45 via immunostaining and flow cytometry of immune and progenitor cells within the synovium, bone marrow, and subchondral bone [36,37], one study which digested subchondral bone chips identified 36% of cells as Cd45+ [38]. Further, large populations of neutrophils (S100a8+, S100a9+, Ly6g+, >25% data not shown) are present within our samples. The mix of developing B-cells and large number of neutrophils suggest a strong component of bone marrow within the samples, as these cells, and other immune cells, arise from marrow [39]. Although it is established that immune cells are the biggest drivers of inflammation in the joint [[40], [41], [42], [43], [44]], the quantity and composition of these cells within the knee has been difficult to discern until now. Our results suggest that the contribution of immune cells to the whole-joint transcriptome may have been underappreciated in previous microarray or bulk RNA-seq studies.

A limitation of this study is that transcription drift is possible during the enzymatic digestions [45]. Bone in particular remains challenging to digest and contains diverse cell populations that are sensitive to transcription drift [46,47]. Even with prior mechanical disruption of bony tissues, extensive enzymatic dissociation is still required [48] and mechanical disruption may lead to its own phenotypic artifacts [[49], [50], [51]]. Due to the necessity of enzymatic digestion of knee tissues at 37^o^C some transcriptional drift is inevitable, as the cells are biologically active at these temperatures, and may increase stress related genes in response to these conditions [52,53]. To reduce phenotypic drift, we minimized the overall time of cells in the digest required for the two-step cell isolation protocol. During all stages that do not require enzymatic digestion, samples were chilled on ice. Neonatal mouse kidneys have been digested using a protease with high activity in the cold, which substantially reduced digestion artifacting, but this has not been demonstrated in more complex and difficult to digest tissues [54]. Inhibition of transcription during digestion is one potential tool shown to reduce this artifacting [53]. Despite this challenge, there is evidence of phenotypic stability in chondrocytes and osteocytes, even with prolonged overnight tissue digestion protocols [[55], [56], [57], [58]]. Cell identification remains a complex challenge, the genes selected are suggestive of broad cell categories and are not meant to provide definitive cell identities. Additionally, the inclusion of circulating blood within the joint and samples may account for a portion of the neutrophils and other immune cells found within the samples. Future work could include an IV perfusion of PBS prior to sacrifice to eliminate these populations in the final samples. The scRNASeq data was generated with a combined eight tissue digests of four stifle joints from two mice. While there is a limitation in the number of biological (mouse) replicates, the two-stage digestion method generated scRNASeq expression data that was reproducible and consistent across all four joints. In future studies, we may consider reducing the effect of biological variability by combining multiple mouse limb digests into a pooled sample prior to library preparation and sequencing. The low number of chondrocytes in the whole-joint relative to other cells could increase the difficulty in determining subtle differences that may exist within these populations. With this approach, there is the potential limitation of missing rarer structural cell-types due to the quantity of immune cells in whole knee digests.

Having established a reproducible protocol for whole joint transcriptomics analysis, our current focus will be on the comparison between joints from different conditions. For example, to study the contributions of individual cell-types in healthy and arthritic joints, sex-related differences, early OA or post-traumatic OA, induced OA models, and the assessment of therapeutic efficacy. Other future work includes the identification of cells using machine learning algorithms [59,60]. Different subtypes of cells, especially macrophages, have demonstrated pro- or anti-inflammatory roles in the joint depending on polarization [[61], [62], [63], [64], [65]]. Transcriptomic data from whole joints could enable the study of immune-stromal cell cross-talk, as these interactions have important implications for OA [66], bone cells [1,67], macrophages [68], synoviocytes [69,70], and NK-cells [71].

In summary, we developed a protocol that reproducibly captures cell-types from soft and hard tissues, generating viable single-cell suspensions and preserving both stromal and immune cells for scRNA-seq. We observed an abundance of immune cells from diverse lineages, as well as chondrocytes and other key structural cells of the knee tissues.

## Authorship Credit

Dustin M. Leale – Wet protocol design, acquisition of data, drafting of the article, interpretation of data, revising intellectual content. Linan Li – Wet protocol design, acquisition of pilot data, Matt Settles – Bioinformatics design and analysis, statistical expertise. Keith Mitchell – Processing of read data, Bioinformatic analysis and toolmaking. Lutz Froenicke - Administrative, technical, or logistic support, sequencing expertise, revising of technical content. Jasper Yik – Interpretation of data, revising intellectual content, administrative, technical, or logistic support. Dominik Haudenschild – Conception of study, design, interpretation of data, revising intellectual content, obtaining of funding, administrative, technical, or logistic support.

## Role of the funding source

This work was supported by the Department of Defense through the CDMRP under Award no. W81XWH1810783 PR171305 and departmental funds to DRH. Funding sources had no involvement in the design of the study, collection, analysis and interpretation of the data or in writing or approving the manuscript. Opinions, interpretations, conclusions, and recommendations are those of the authors and are not necessarily endorsed by the Department of Defense.

## Conflict of interest statement

No commercial funding was received for the work. The University of California has patents and pending patents on some of the findings, with Drs. Haudenschild and Yik listed as inventors. Drs. Haudenschild and Yik are co-founders of Tesio Pharmaceuticals, Inc, which is in the process of licensing related intellectual property from The University of California.
